# Evaluation of PD-L1 Expression in Undifferentiated Pleomorphic Sarcomas, Liposarcomas and Chondrosarcomas

**DOI:** 10.3390/biom12020292

**Published:** 2022-02-11

**Authors:** Yifan Zhang, Yi Chen, Andri Papakonstantinou, Panagiotis Tsagkozis, Christina Linder-Stragliotto, Felix Haglund

**Affiliations:** 1 Department of Pathology and Cancer Diagnostics, Radiumhemmet, Karolinska University Hospital Solna, 171 64 Solna, Sweden; felix.haglund@ki.se; 2 Department of Oncology-Pathology, Karolinska Institutet, 171 77 Solna, Sweden; yi.chen@ki.se (Y.C.); andri.papakonstantinou@ki.se (A.P.); 3 Department of Breast Cancer, Endocrine Tumors and Sarcomas, Karolinska University Hospital, 171 64 Solna, Sweden; christina.linder-stragliotto@regionstockholm.se; 4 Department of Molecular Medicine and Surgery, Karolinska Institutet, 171 77 Solna, Sweden; panagiotis.tsagkozis@ki.se; 5 Department of Orthopedics, Karolinska University Hospital, 171 64 Solna, Sweden

**Keywords:** sarcoma, immunotherapy, PD-L1, chondrosarcoma, liposarcoma, undifferentiated pleomorphic sarcoma

## Abstract

Immune checkpoint inhibitors (ICIs) such as PD1/PD-L1 blockers are an established treatment for many solid cancers. There are currently no approved ICIs for sarcomas, but satisfactory results have been seen in some patients with disseminated disease in certain histological types. Most studies on PD-L1 in sarcoma have used small specimens and there are no clear cutoff values for scoring. We investigated PD-L1 immunoreactivity in high-grade chondrosarcomas (CS), abdominal liposarcoma (LS) and undifferentiated pleomorphic sarcomas (UPS). In total, 230 tumors were stained with SP142 and SP263 assays and evaluated by two clinical pathologists. Immunoreactivity in tumor and immune cells was correlated with clinical outcome. Overall, ≥1% PD-L1 immunoreactivity in tumor cells was found in 11 CS, 26 LS and 59 UPS (SP142 assay) and in 10 CS, 26 LS and 77 UPS (SP263 assay). Most tumors exhibited ≤10% PD-L1 immunoreactivity, but a subset across all three subtypes had >50%. Kaplan–Meier survival analysis showed no significant difference in metastasis-free or overall survival in relation to PD-L1 immunoreactivity in tumor or immune cells for any subtype. As there is a lack of clinical data regarding PD-L1/PD-1 status and therapy response, it is not currently possible to establish clear cutoff values. Patients with high (>50%) PD-L1 immunoreactivity in tumor cells (TC) with the SP263 assay would be a logical group to investigate for potentially beneficial PD1/PD-L1-targeted treatment.

## 1. Introduction

The complex network of interactions present in the tumor microenvironment has in recent years risen, as the new frontier of targeted cancer therapies for a number of malignant tumors, with the programmed cell death protein 1 (PD-1)/programmed cell death ligand 1 (PD-L1) pathway as the currently most widely utilized target. PD-L1 is a type 1 transmembrane protein, and a ligand to PD-1, a receptor found on T, B and myeloid cells. The binding of PD-1 and PD-L1 suppresses the adaptive immune system by interactions with phosphatases (SHP-1 or -2) via ITSM (Immunoreceptor Tyrosine-based Switch Motif [1,2]. Tumor cells utilize this mechanism to escape the immune system, which in turn, by inhibiting this bond, may properly identify and attack tumor cells. PD-L1 immunoreactivity is now part of routine workup in many solid cancers, but is not yet established in sarcomas. 

Targeted treatments using immune checkpoint inhibitors (ICI) blocking the binding between PD-L1 and PD1 is already established in many solid cancers such as melanoma and lung cancer, with favorable response even in inoperable or disseminated disease [3,4]. Treatment response is often correlated with the presence of tumor-infiltrating lymphocytes (TILs) and PD-L1 expression in both tumor and immune cells [1,5]. Association with worse clinical outcome has been demonstrated in solid cancers [6,7,8] as well as soft tissue sarcomas and osteosarcoma [9,10,11]. Other studies have found PD-L1 expression to be a positive predictive marker [11], or that it had no significant impact on prognosis [12,13,14,15]. 

The diversity and rarity of sarcomas pose challenges; sample sizes are often small and difficult to extrapolate across subtypes. Current studies show significant variation in both PD-L1 immunoreactivity and blockade efficacy among histological subtypes, which seem to be the most important factors in that regard [16]. Sarcomas found to have PD-L1 immunoreactivity include, among others, angiosarcoma, chondrosarcoma, osteosarcoma, Ewing’s sarcoma, liposarcoma, rhabdomyosarcoma, synovial sarcoma [7], undifferentiated pleomorphic sarcoma and MPNST [16,17,18,19,20]. Currently, there are no ICIs formally approved for treatment of sarcomas, although it can be considered for specific soft tissue sarcoma subtypes based on results from phase II studies [21].

Though cutoff values have been defined in some cancers such as non-small-cell lung cancer, the predictive and prognostic values of PD-L1 expression have not yet reached a consensus in many other cancer types. There are clear protocols for scoring PD-L1 in cancers with formal indication for ICI with cutoff values depending on histological type as well as antibody assay. The lack of established protocols for evaluating PD-L1 immunohistochemistry staining in sarcomas is a major challenge, given that different histological subtypes might have different cutoff values. Existing studies often adapt pre-existing protocols [22,23], but the absence of consensus results in interstudy variation in cutoffs for positive expression. 

In this study, we aimed to categorically investigate and quantify PD-L1 immunoreactivity in three major sarcoma subtypes; chondrosarcoma (CS), liposarcoma (LS) and undifferentiated pleomorphic sarcoma (UPS), using the SP142 and SP263 assays, which are two of the most used in vitro diagnostic regulation (IVDR)-approved PD-L1 immunohistochemistry assays used in clinical settings.

## 2. Materials and Methods

### 2.1. Patients and Tumor Samples

The study was based on three previously published cohorts of primary chondrosarcoma (CS) [24], abdominal liposarcomas (LS) and undifferentiated pleomorphic sarcomas (UPS) and patient and tumor characteristics were previously reported [25]. Since grade 1 chondrosarcoma has very limited metastatic potential, these were excluded from the analysis. All cases were identified through the archives of the Department of Clinical Pathology and Cytology at Karolinska University Hospital from 1994–2020. We excluded grade 1 chondrosarcomas from the CS cohort since these tumors had very low malignant potential. Clinical data were retrieved from the digital patient records and was available for all patients.

### 2.2. TCGA Gene Expression Data

Both gene expression data (LS, *n* = 58; UPS, *n* = 50) and corresponding clinical data were obtained through the TCGA sarcoma (SARC) cohort from UCSC Xena (http://xena.ucsc.edu/ accessed on 26 February 2021) website. PD-L1 expression was based on transcriptome levels based on RNA-seq. Among all cases, the scaled estimate values from RNA-seq by Expectation Maximization (RSEM) were log2 transformed and converted to transcripts per million (TPM) values by multiplying by one million. For grouping by PD-L1 status, we determined the cutoff as the median value of CD274 gene expression level as a surrogate of PD-L1.

### 2.3. Immunohistochemistry and Scoring

Immunohistochemistry was performed on full slides using Ventana PD-L1 (SP142) and Ventana PD-L1 (SP263) assays (Roche, Basel, Schweiz). The staining procedure was performed at a clinically certified laboratory (SWEDAC accreditation) at the Department of Clinical Pathology and Cytology, Karolinska University Hospital according to instructions from the manufacturer (Ventana Benchmark ULTRA, Roche, Basel, Schweiz). Each staining batch included one positive and one negative control.

The slides were independently evaluated by two pathologists in a consensus manner. No specific pre-existing protocol was adapted during scoring. Immune cells (IC) were noted if present and graded 1–3 depending on infiltrate density (1—focal, 2—intermediate, 3—dense). The individual cell types in the inflammatory infiltrates were not further characterized. The amount of tumor cells (TC) and immune cells (IC) with PD-L1 immunoreactivity were estimated to the nearest percent and sorted into 4 categories for LS and UPS: none (0%), low (<5%), intermediate (5–9%) and high (≥10%). PD-L1 immunoreactivity for CS was sorted into only two categories: negative (0%) and positive (≥1%).

### 2.4. Ethical Permission

The study was approved by the local ethical board (study registration number 2013 1979-31, approval on 15 January 2014) and all patients had given informed consent in accordance with the Swedish biobank law. 

### 2.5. Statistics

Time to event was defined as the timeframe between the date of surgery and the date of the event, defined either as first known metastasis or death. Differences in overall (OS)- and metastasis-free survival (MFS) were calculated using the log-rank method, and the differences were plotted on Kaplan–Meier curves. A two-sided Fisher’s exact test and chi-squared test were used to compare categorical variables. A *p*-value of <0.05 was defined as statistically significant. Correlation between the SP142 and SP263 assays were calculated using Pearson correlation test. To ascertain the association between clinical features, PD-L1 progression and prognosis in CS, LS and UPS univariate analysis was performed using the Cox regression model in R package “survival”. 

## 3. Results

### 3.1. Clinical Data

A total of 230 tumors from 214 patients were included: 76 tumors from 74 patients with chondrosarcoma, 58 tumors from 44 patients with liposarcoma and 96 tumors from 96 patients with undifferentiated pleomorphic sarcoma. Of the 96 patients with UPS, 18 cases were included based on ad hoc staining of PD-L1 (SP263) in the clinical setting and were not stained with SP142. The average age was 58 (range 16–83) years for CS, 69 (26–86) for LS and 70 (46–93) for UPS. Median overall survival was 11 (0,3–39) years for CS, 4 (0,1–32) for LS and 3 (0,1–12) for UPS. None of the patients had received systemic therapy with ICIs or other immune-modulating agents.

### 3.2. Chondrosarcoma

**Figure 1:**
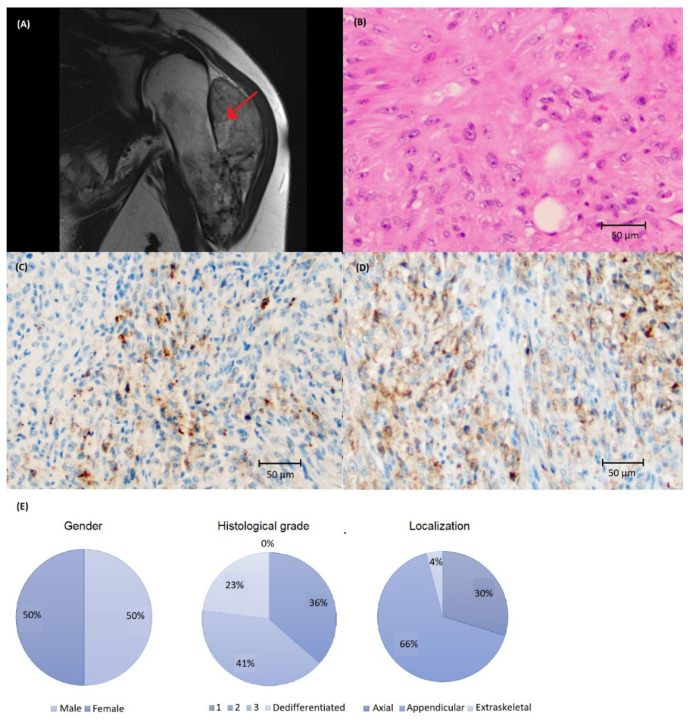
(**A**) Magnetic resonance imaging depicting chondrosarcoma (CS) in the humerus (arrow) and (**B**) histology of high-grade CS stained with (**C**) SP142 and (**D**) SP263 assays at ×200 magnification showing a higher number of cells with PD-L1 immunoreactivity using the SP263 assay. (**E**) Clinical and tumor characteristics of the CS cohort.

**Figure A1:**
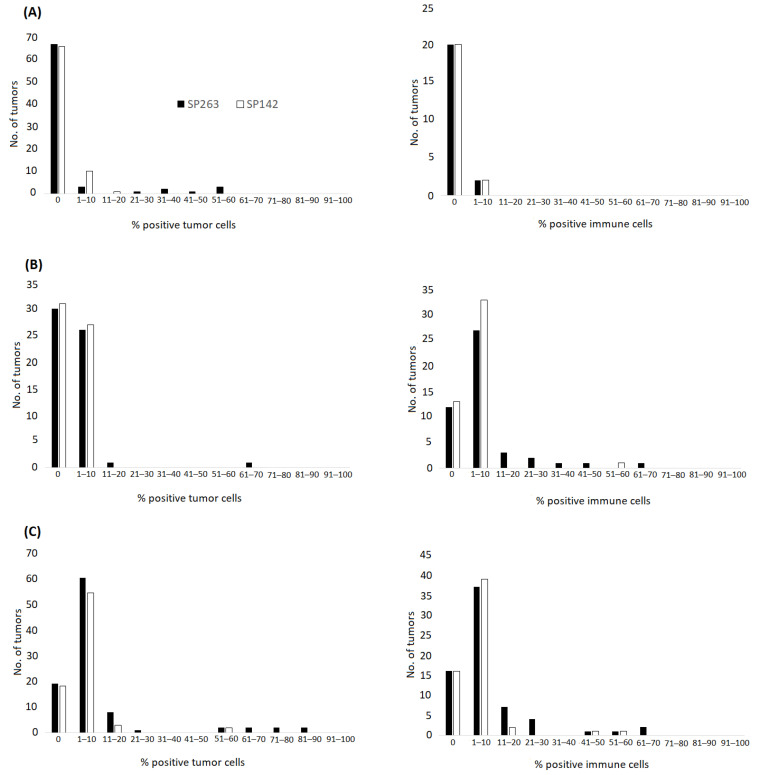
Distribution of PD-L1 immunoreactivity in tumor and immune cells with SP142 and SP263 assays for (**A**) chondrosarcoma, (**B**) liposarcoma and (**C**) undifferentiated pleomorphic sarcoma.

Clinical characteristics and visualization of PD-L1 staining is demonstrated in Figure 1. The majority of CS were PD-L1-negative (Table 1). PD-L1 immunoreactivity was more common in tumors with higher histological grade (grade 3 or dedifferentiated) and not observed in any grade 2 tumors. Three tumors also showed >50% immunoreactivity in TC using SP263 (Figure A1). The presence of immune cells (IC) was rare; 22 cases had IC present, and only two cases had ≥1% immunoreactivity (Table 1, Figure A1).

**Figure 2:**
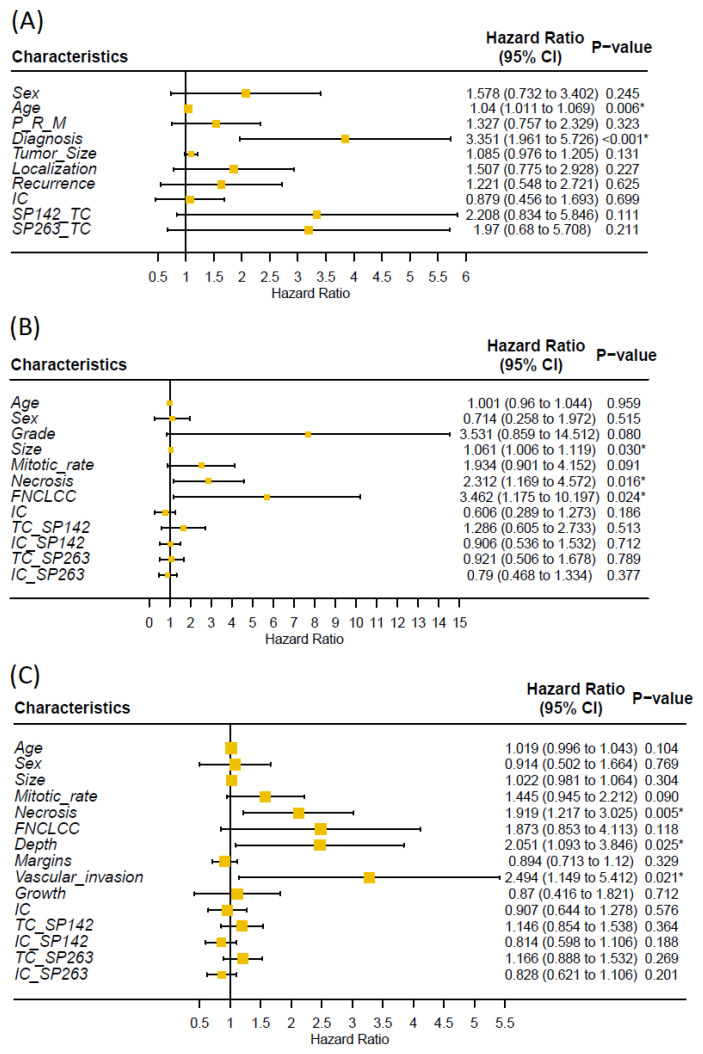
Univariate cox regression analysis of association between clinical/tumor characteristics, PD-L1 immunoreactivity and overall survival of (**A**) chondrosarcoma, (**B**) liposarcoma and (**C**) undifferentiated pleomorphic sarcoma (P = primary, M = metastasis, R = recurrence, * = *p* < 0.05). We found no significant association between PD-L1 immunoreactivity and overall survival.

**Figure 3:**
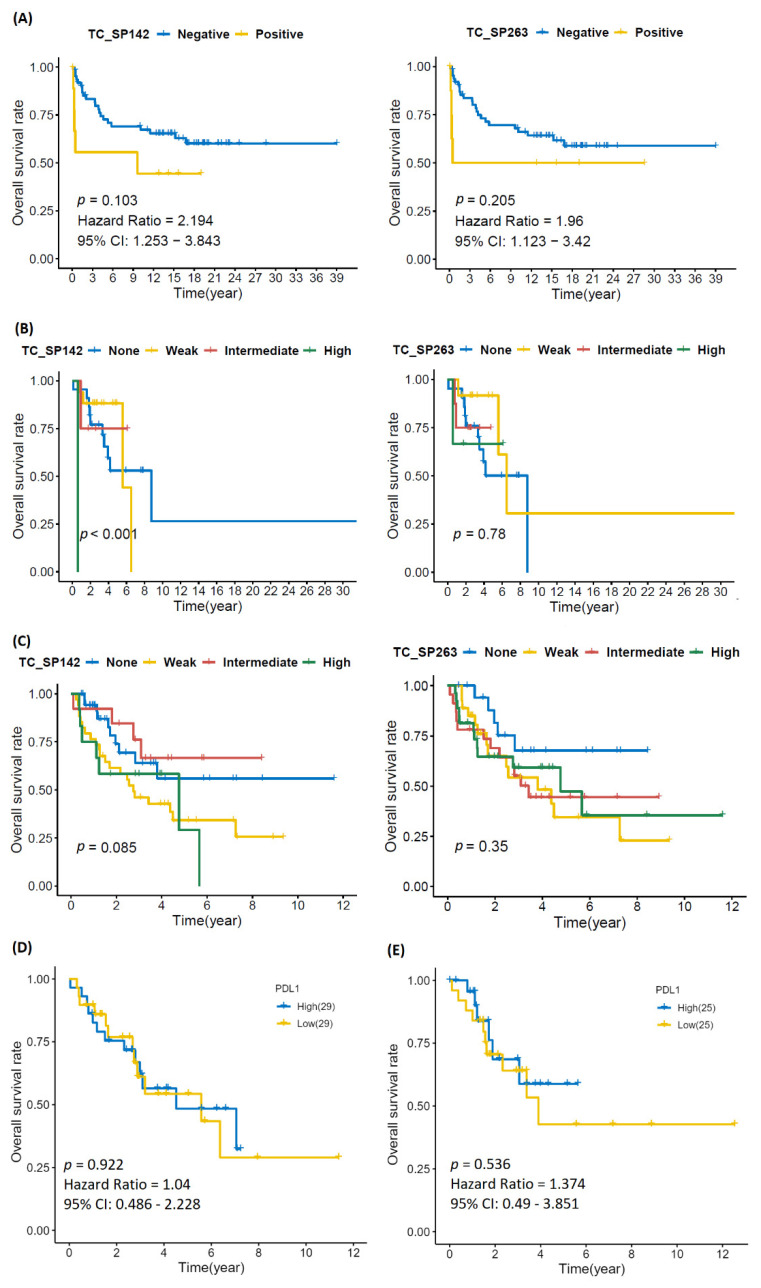
Kaplan–Meier curves depicting overall survival in (**A**) chondrosarcoma, (**B**) liposarcoma and (**C**) undifferentiated pleomorphic sarcoma in relation to PD-L1 immunoreactivity in tumor cells, as well as data from the Cancer Genome Atlas for (**D**) LS and (**E**) UPS, showing no significant impact of PD-L1 status on overall survival. Kaplan–Meier survival analysis was performed by log-rank test.

**Figure A2:**
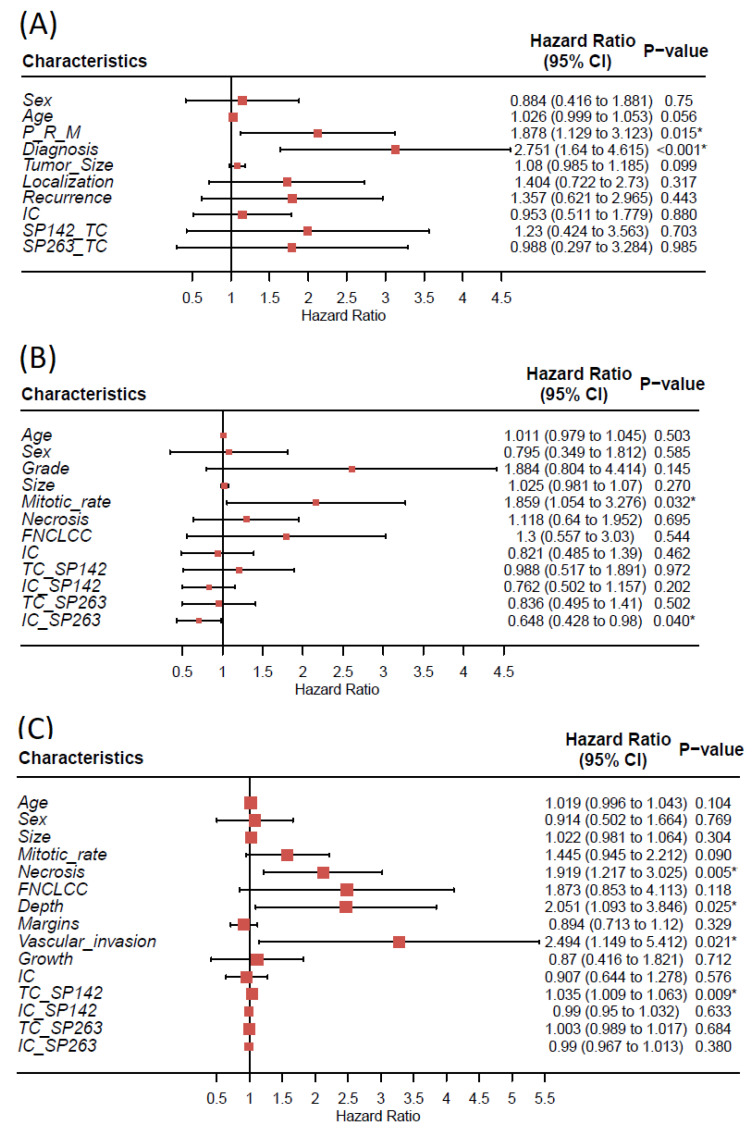
Univariate analysis of association between clinical/tumor characteristics, PD-L1 immunoreactivity and metastasis-free survival (MFS) of (**A**) chondrosarcoma, (**B**) liposarcoma and (**C**) undifferentiated pleomorphic sarcoma (* = *p* < 0.05).

Univariate analysis showed that higher age and tumor grade were significantly associated with shorter MFS and OS (Figure 2 and Figure A2). There was no significant correlation between PD-L1 immunoreactivity and clinical outcome; Kaplan–Meier analysis showed that patients with PD-L1-positive tumors had shorter OS, but the difference was nonsignificant and most likely explained by the association to high tumor grade (Figure 3).

### 3.3. Liposarcoma

**Figure 4:**
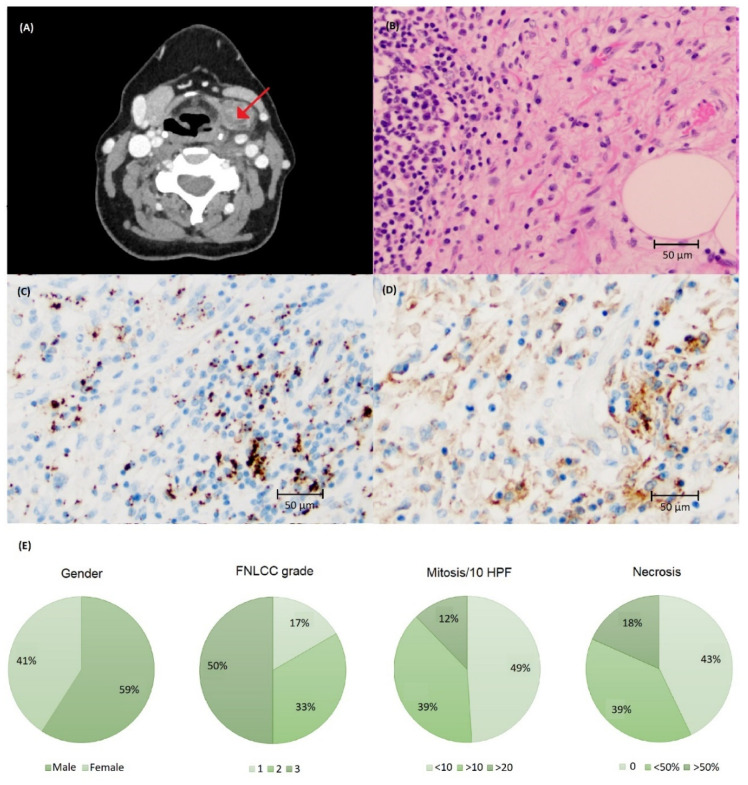
(**A**) Computed tomography of liposarcoma (LS) in the neck region (arrow). (**B**) Histology of LS stained with (**C**) SP142 and (**D**) SP263 assays at ×200 magnification showing a higher number of cells with PD-L1 immunoreactivity using the SP263 assay. (**E**) Clinical and tumor characteristics of the LS cohort.

Clinical characteristics and visualization of PD-L1 staining is demonstrated in Figure 4. Most tumors had either none or low immunoreactivity with SP142 and none, low or intermediate with SP263 (Table 1). One tumor had >50% PD-L1 immunoreactivity in TC using SP263 (Figure A1). Immune infiltrate was present in 48 cases; 35 cases expressed ≥1% immunoreactivity with SP263 and 34 cases with SP142 (Table 1, Figure A1).

**Figure A3:**
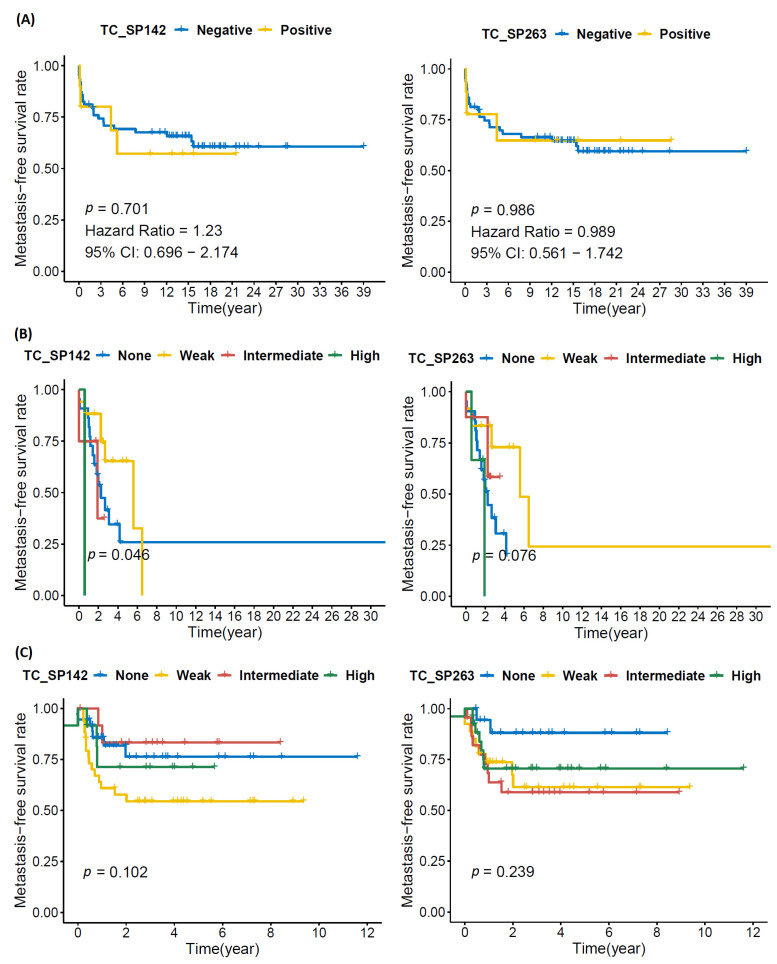
Kaplan–Meier curves depicting MFS based on PD-L1 immunoreactivity in tumor cells for (**A**) chondrosarcoma, (**B**) liposarcoma and (**C**) undifferentiated pleomorphic sarcoma.

**Figure A4:**
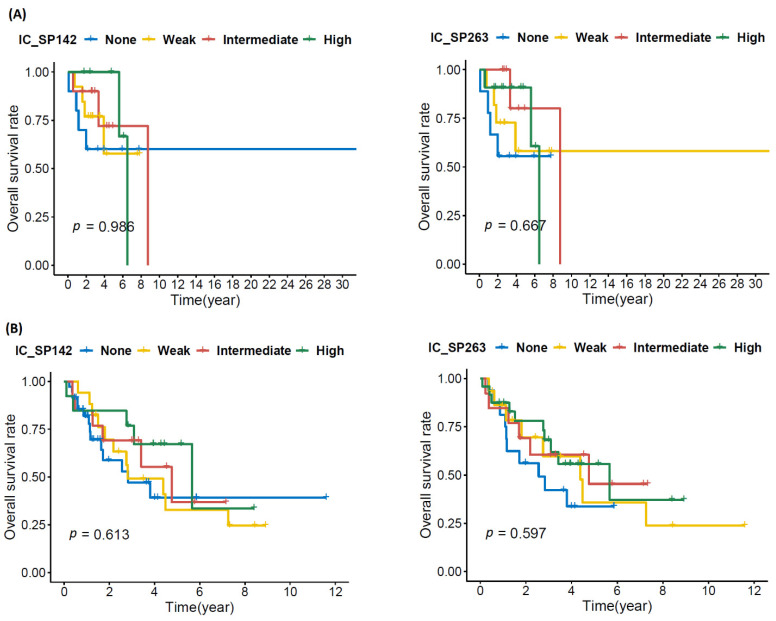
Kaplan–Meier curves depicting overall survival (OS) based on PD-L1 immunoreactivity in immune cells for (**A**) liposarcoma and (**B**) undifferentiated pleomorphic sarcoma.

**Figure A5:**
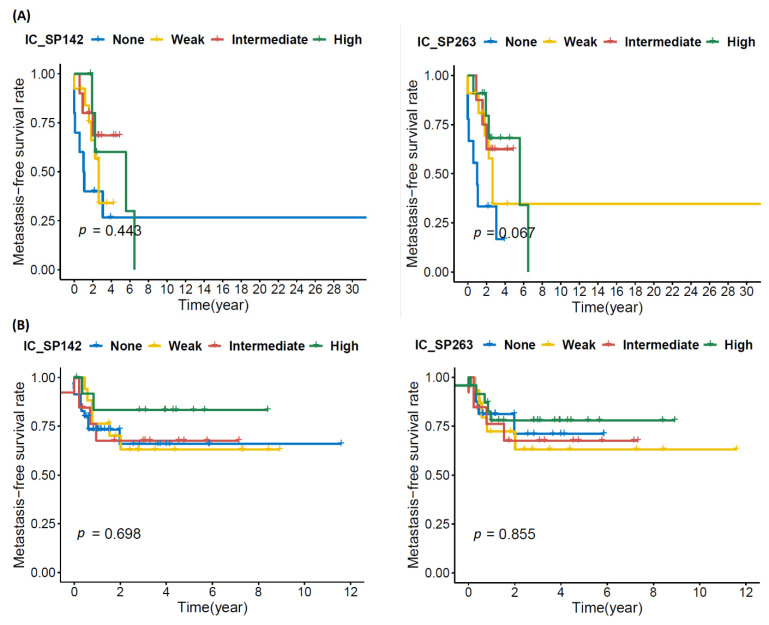
Kaplan–Meier curves depicting MFS based on PD-L1 immunoreactivity in immune cells for (**A**) liposarcoma and (**B**) undifferentiated pleomorphic sarcoma.

Univariate analysis showed that the presence of necrosis and higher FNLCC grade were significant associated with shorter MFS and OS (Figure 2 and Figure A2). Kaplan–Meier showed a statistically significant reduction in MFS and OS in patients with PD-L1 immunoreactivity for the SP142 assay, but not for SP263; however, no statistically significant impact was seen using univariate analysis (Figure 2, Figure 3 and Figure A3). PD-L1 immunoreactivity in IC had no significant impact on MFS and OS (Figure A4 and Figure A5).

### 3.4. Undifferentiated Pleomorphic Sarcoma

**Figure 5:**
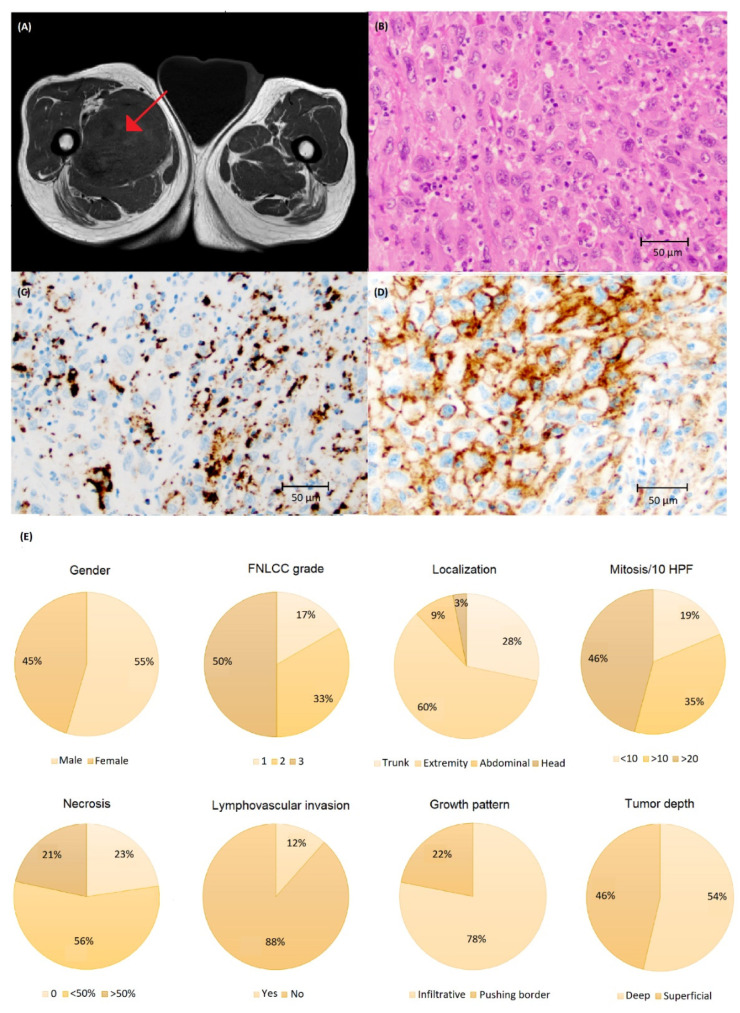
(**A**) Magnetic resonance imaging of undifferentiated pleomorphic sarcoma (UPS) in the thigh (arrow) and (**B**) histology of UPS stained with (**C**) SP142 and (**D**) SP263 assays at ×200 magnification showing a higher number of cells with PD-L1 immunoreactivity using the SP263 assay. (**E**) Clinical and tumor characteristics of the UPS cohort.

Clinical characteristics and visualization of PD-L1 staining are demonstrated in Figure 5. PD-L1 immunoreactivity was more common in UPS (Table 1) compared to CS and LS (*p* < 0.05). Furthermore, >50% immunoreactivity was seen in two and eight tumors using the SP142 and SP263 assay, respectively (Figure A1). Immune cells were observed in 68 cases with 43 and 52 cases expressing ≥1% immunoreactivity with SP142 and SP263, respectively (Table 1, Figure A1).

Univariate analysis showed that the presence of necrosis, tumor growth in deep tissue and vascular invasion were significantly associated with shorter MFS and OS (Figure 2 and Figure A2). Kaplan–Meier showed no significant difference in MFS and OS in patients with PD-L1-positive vs. -negative TC or IC (Figure 3, Figure A3, Figure A4 and Figure A5).

### 3.5. Correlation between SP142 and SP263 Assays

Pearson’s coefficient test showed a moderately positive correlation between SP142 and SP263 arrays in tumor cells for chondrosarcoma (r = 0.62, *p* < 0.05) and undifferentiated pleomorphic sarcoma (r = 0.74, *p* < 0.05), and a strong positive correlation for liposarcoma (r = 0.83, *p* < 0.05).

### 3.6. The Cancer Genome Atlas

Gene expression data were extracted from The Cancer Genome Atlas (TCGA) on LS and UPS in order to investigate any correlation between PD-L1 gene expression and clinical outcome. Kaplan–Meier curves showed no significant difference in MFS or OS between PD-L1 positive and negative cases for any of the two sarcoma subtypes (Figure 3).

## 4. Discussion

In this study, we aimed to investigate if positive expression of PD-L1 in tumor and immune cells was associated with prognosis in three sarcoma subtypes using commercially available assays approved for diagnostic use. In addition, we aimed to elucidate if any clear cutoff for prognostic subtyping based on PD-L1 immunoreactivity could be derived from the data.

In our cohort of 214 patients, we found that PD-L1 immunoreactivity in TC and IC was overall negative or low (<10%) in CS and LS. PD-L1 immunoreactivity was more commonly observed in UPS, in line with previous publications [10,26]. A subset of tumors in all three subtypes had high (>50%) PD-L1 expression in TC and IC, especially using the SP263 assay, which generally detected a higher % of PD-L1-positive cells, both TC and IC. There was a strong concordance between the two assays regarding the number of tumors with PD-L1 reactivity. This is in line with results from previous studies, which have shown that the SP142 assay generally stains a lower number of both tumor and immune cells [27,28].

Our cutoffs for statistical analysis were set at 0%, <5%, 5–9% and ≥10% for none, low, intermediate and high PD-L1 immunoreactivity for UPS and LS, respectively; for CS the cutoffs were set at 0% and ≥1 for negative and positive, respectively. These values were chosen as we aimed exclude tumors with only a few positive cells and to identify those patients with “true” PD-L1-positive tumors who may have a satisfactory response to ICIs in a clinical setting in some solid tumors. It is, however, worth noting that PD-L1-negative sarcomas have also shown satisfactory response, albeit to a lesser extent [29].

We found no significant difference in MFS and OS in all three sarcoma subtypes regardless of PD-L1 expression. In CS there was slightly worse, but not statistically significant, MFS and OS in patients with positive PD-L1 expression; however, this is likely due to the fact that PD-L1 immunoreactivity was only observed in grade 3 or dedifferentiated tumors. In a meta-analysis, Zheng et al. found PD-L1 immunoreactivity to be a negative prognostic factor in soft tissue sarcomas [8], while other studies found no significant impact on survival in retroperitoneal liposarcoma [30] and chondrosarcoma [12,14]. While the practical utility of PD-L1 as a prognostic marker may be limited, the true value of this marker must be assessed in the context of response to ICIs (which is outside the scope of this study). Worth noting is also the dynamic nature of PD1/PD-L1 and discordance of PD-L1 expression in primary versus metastatic tumors, which has been observed in both solid tumors and sarcomas [31,32,33], and its role as a predictive biomarker may require assessment of more than one tumor from the patient. As it stands currently, none of the patients had received ICIs and hence the lack of clinical data is a major limiting factor to drawing absolute conclusions on the clinical impact of PD-L1 in this patient group.

While there are no approved ICIs for the treatment of sarcomas, there are several phase II studies, although the rarity and heterogeneity of sarcomas pose a challenge for conducting phase III trials. SARC028 studied the effects of Pembrolizumab; objective response was found in 7 of 40 patients with soft tissue sarcoma, with 4 being patients with UPS, and in 2 of 40 patients with bone sarcoma. Only three patients in total had PD-L1-positive (≥1%) tumors, all of them with UPS [34]. In Alliance A091401 [29], 2 of 43 patients responded to single-therapy with Nivolumab and 6 of 42 responded to Nivolumab + Ipilimumab; two of these patients had UPS, and other subtypes included leiomyosarcoma, alveolar soft part sarcoma, angiosarcoma and myofibrosarcoma. PD-L1 statuses of included patients were not reported. Toulmonde et al. [13] found that only 3 of 50 patients with soft tissue sarcoma showed any response to treatment with Pembrolizumab and Cyclophosphamide; six patients in total had a PD-L1-positive (≥1%) tumor. No patients with UPS had a satisfactory response in PEMBROSARC [35], but only one of those had a PD-L1-positive (≥1%) tumor. The recent international recommendation for treatment of sarcomas using ICIs suggests that ICIs may be considered in a few sarcoma subtypes where first-line therapy has given unsatisfactory results, such as UPS, alveolar soft-part sarcoma, cutaneous angiosarcoma and classic Kaposis’ sarcoma [21,36]. 

In conclusion, our cohort found no association between PD-L1 immunoreactivity and prognosis in CS, LS or UPS, with PD-L1 immunoreactivity being rare in CS and LS but more common in UPS. The SP142 assay stained less cells overall and cannot be used interchangeably with SP263. Previous studies have shown satisfactory response in some patients, and those with PD-L1 immunoreactivity could profit from PD1/PD-L1 targeted treatment. Since we lack data with regard to clinical response to PD1/PD-L1 inhibitors, it is not possible to establish a cutoff value for PD-L1 immunoreactivity in these sarcoma subtypes. However, in analogy with the staining patterns found in carcinomas, we distinguished a subset of cases with definitive high PD-L1 expression. Future clinical studies on ICI in sarcomas should take these findings into account, since enrolling a sufficient number of patients with high tumor PD-L1 expression would require multicenter clinical studies. Nevertheless, establishment of working ICI biomarkers could be of great value for sarcoma patients, even though only a smaller portion of patients might benefit from the treatment. Investigating other biomarkers such as tumor mutational burden and mismatch repair deficiency may also be of prognostic and predictive importance. As PD-L1 immunohistochemistry is widely established, it should be performed on tumors from sarcoma patients with disseminated disease and unsatisfactory response from first line treatment. 

## Figures and Tables

**Table 1:** PD-L1 immunoreactivity in chondrosarcomas, liposarcomas and undifferentiated pleomorphic sarcomas stained with SP142 and SP263 immunoassays (Roche).

Sarcoma type	PD-L1 Immunoreactivity	TC (No. of Cases)		IC (No. of Cases)	
		SP142	SP263	SP142	SP263
Chondrosarcoma					
	0	65	66	20	20
	>1%	11	10	2	2
Liposarcoma					
	0	31	30	13	12
	<5%	22	15	15	13
	5–9%	4	9	13	8
	≥10%	1	4	6	14
Undifferentiated pleomorphic sarcoma					
	0	18	19	16	16
	<5%	34	27	17	15
	5–9%	13	23	13	13
	≥10%	12	27	12	24

## Data Availability

Not applicable.

## References

[1] LaFleur M.W., Muroyama Y., Drake C.G., Sharpe A.H. (2018). Inhibitors of the PD-1 Pathway in Tumor Therapy. J. Immunol..

[2] Thanindratarn P., Dean D.C., Nelson S.D., Hornicek F.J., Duan Z. (2019). Advances in immune checkpoint inhibitors for bone sarcoma therapy. J. Bone Oncol..

[3] Mahoney K.M., Freeman G.J., McDermott D.F. (2015). The Next Immune-Checkpoint Inhibitors: PD-1/PD-L1 Blockade in Melanoma. Clin Ther..

[4] Geng Y., Zhang Q., Feng S., Li C., Wang L., Zhao X., Yang Z., Li Z., Luo H., Liu R. (2021). Safety and Efficacy of PD-1/PD-L1 inhibitors combined with radiotherapy in patients with non-small-cell lung cancer: A systematic review and meta-analysis. Cancer Med..

[5] Yi M., Jiao D., Xu H., Liu Q., Zhao W., Han X., Wu K. (2018). Biomarkers for predicting efficacy of PD-1/PD-L1 inhibitors. Mol. Cancer.

[6] Ohigashi Y., Sho M., Yamada Y., Tsurui Y., Hamada K., Ikeda N., Mizuno T., Yoriki R., Kashizuka H., Yane K. (2005). Clinical Significance of Programmed Death-1 Ligand-1 and Programmed Death-1 Ligand-2 Expression in Human Esophageal Cancer. Clin. Cancer Res..

[7] Massi D., Brusa D., Merelli B., Ciano M., Audrito V., Serra S., Buonincontri R., Baroni G., Nassini R., Minocci D. (2014). PD-L1 marks a subset of melanomas with a shorter overall survival and distinct genetic and morphological characteristics. Ann. Oncol..

[8] Xiang X., Yu P.-C., Long D., Liao X.-L., Zhang S., You X.-M., Zhong J.-H., Li L.-Q. (2017). Prognostic value of PD-L1 expression in patients with primary solid tumors. Oncotarget.

[9] Zheng C., You W., Wan P., Jiang X., Chen J., Zheng Y., Li W., Tan J., Zhang S. (2018). Clinicopathological and prognostic significance of PD-L1 expression in sarcoma: A systematic review and meta-analysis. Medicine.

[10] Bertucci F., Finetti P., Perrot D., Leroux A., Collin F., Le Cesne A., Coindre J.-M., Blay J.-Y., Birnbaum D., Mamessier E. (2017). PDL1 expression is a poor-prognosis factor in soft-tissue sarcomas. OncoImmunology.

[11] Kim J.R., Moon Y.J., Kwon K.S., Bae J.S., Wagle S., Kim K.M., Park H.S., Lee H., Moon W.S., Chung M.J. (2013). Tumor infiltrating PD1-positive lymphocytes and the expression of PD-L1 predict poor prognosis of soft tissue sarcomas. PLoS ONE.

[12] Matikas A., Zerdes I., Lövrot J., Richard F., Sotiriou C., Bergh J., Valachis A., Foukakis T. (2019). Prognostic Implications of PD-L1 Expression in Breast Cancer: Systematic Review and Meta-analysis of Immunohistochemistry and Pooled Analysis of Transcriptomic Data. Clin. Cancer Res..

[13] Toulmonde M., Adam J., Bessede A., Ranchère-Vince D., Velasco V., Brouste V., Blay J.-Y., Mir O., Italiano A. (2016). Integrative assessment of expression and prognostic value of PDL1, IDO, and kynurenine in 371 primary soft tissue sarcomas with genomic complexity. J. Clin. Oncol..

[14] Botti G., Scognamiglio G., Marra L., Pizzolorusso A., Di Bonito M., De Cecio R., Cantile M., De Chiara A. (2017). Programmed Death Ligand 1 (PD-L1) Expression in Primary Angiosarcoma. J. Cancer.

[15] Kostine M., Cleven A.H., de Miranda N., Italiano A., Cleton-Jansen A.-M., Bovee J. (2016). Analysis of PD-L1, T-cell infiltrate and HLA expression in chondrosarcoma indicates potential for response to immunotherapy specifically in the dedifferentiated subtype. Mod. Pathol..

[16] Zuo W., Zhao L. (2019). Recent advances and application of PD-1 blockade in sarcoma. OncoTargets Ther..

[17] Keung E.Z., Tsai J.W., Ali A.M., Cormier J.N., Bishop A.J., Guadagnolo B.A., Torres K.E., Somaiah N., Hunt K.K., Wargo J.A. (2018). Analysis of the immune infiltrate in undifferentiated pleomorphic sarcoma of the extremity and trunk in response to radiotherapy: Rationale for combination neoadjuvant immune checkpoint inhibition and radiotherapy. Oncoimmunology.

[18] Park H.K., Kim M., Sung M., Lee S.E., Kim Y.J., Choi Y.-L. (2018). Status of programmed death-ligand 1 expression in sarcomas. J. Transl. Med..

[19] Veenstra R., Kostine M., Cleton-Jansen A.-M., de Miranda N., Bovée J.V. (2017). Immune checkpoint inhibitors in sarcomas: In quest of predictive biomarkers. Lab. Investig..

[20] Kosemehmetoglu K., Ozogul E., Babaoglu B., Tezel G.G., Gedikoglu G. (2017). Programmed death ligand 1 (pd-l1) expression in malignant mesenchymal tumors. Turk. J. Pathol..

[21] Gronchi A., Miah A.B., Dei Tos A.P., Abecassis N., Bajpai J., Bauer S., Biagini R., Bielack S., Blay J.Y., Bolle S. (2021). Soft tissue and visceral sarcomas: ESMO-EURACAN-GENTURIS Clinical Practice Guidelines for diagnosis, treatment and follow-up. Ann Oncol..

[22] Kim C., Kim E.K., Jung H., Chon H.J., Han J.W., Shin K.-H., Hu H., Kim K.S., Choi Y.D., Kim S. (2016). Prognostic implications of PD-L1 expression in patients with soft tissue sarcoma. BMC Cancer.

[23] Kelany M.R., Barth T., Salem D., Mosaad M. (2019). PD-L1 expression in soft tissue sarcomas and its prognostic implication. J. Clin. Oncol..

[24] Zhang Y., Chen Y., Yang C., Seger N., Hesla A.C., Tsagkozis P., Larsson O., Lin Y., Haglund F. (2021). TERT promoter mutation is an objective clinical marker for disease progression in chondrosarcoma. Mod. Pathol..

[25] Su Y., Tsagkozis P., Papakonstantinou A., Tobin N., Gultekin O., Malmerfelt A., Ingelshed K., Neo S., Lundquist J., Chaabane W. (2021). CD11c-CD8 Spatial Cross Presentation: A Novel Approach to Link Immune Surveillance and Patient Survival in Soft Tissue Sarcoma. Cancers.

[26] Boxberg M., Steiger K., Lenze U., Rechl H., von Eisenhart-Rothe R., Wörtler K., Weichert W., Langer R., Specht K. (2018). PD-L1 and PD-1 and characterization of tumor-infiltrating lymphocytes in high grade sarcomas of soft tissue—prognostic implications and rationale for immunotherapy. OncoImmunology.

[27] Hendry S., Byrne D.J., Wright G.M., Young R.J., Sturrock S., Cooper W.A., Fox S.B. (2018). Comparison of Four PD-L1 Immunohistochemical Assays in Lung Cancer. J. Thorac. Oncol..

[28] Oyan B., Sonmez O., Yazar A., Teomete M. (2018). Comparison of SP142 (Ventana) and SP263 (Ventana) assays to test PD-L1 ex-pression in metastatic cancer patients to be treated with immune checkpoint inhibitors. J. Clin. Oncol..

[29] Italiano A., Bellera C., D’Angelo S. (2020). PD1/PD-L1 targeting in advanced soft-tissue sarcomas: A pooled analysis of phase II trials. J. Hematol. Oncology..

[30] Yan L., Wang Z., Cui C., Guan X., Dong B., Zhao M., Wu J., Tian X., Hao C. (2019). Comprehensive immune characterization and T-cell receptor repertoire heterogeneity of retroperitoneal liposarcoma. Cancer Sci..

[31] Li M., Li A., Zhou S., Xu Y., Xiao Y., Bi R., Yang W. (2018). Heterogeneity of PD-L1 expression in primary tumors and paired lymph node metastases of triple negative breast cancer. BMC Cancer.

[32] Rozenblit M., Huang R., Danziger N., Hegde P., Alexander B., Ramkissoon S., Blenman K., Ross J.S., Rimm D.L., Pusztai L. (2020). Comparison of PD-L1 protein expression between primary tumors and metastatic lesions in triple negative breast cancers. J. Immunother. Cancer.

[33] Vargas A.C., MacLean F.M., Sioson L., Tran D., Bonar F., Mahar A., Cheah A.L., Russell P., Grimison P., Richardson L. (2020). Prevalence of PD-L1 expression in matched recurrent and/or metastatic sarcoma samples and in a range of selected sarcomas subtypes. PLoS ONE.

[34] Tawbi H.A., Burgess M., Bolejack V., Van Tine B.A., Schuetze S.M., Hu J., D’Angelo S., Attia S., Riedel R.F., Priebat D.A. (2017). Pembrolizumab in advanced soft-tissue sarcoma and bone sarcoma (SARC028): A multicentre, two-cohort, single-arm, open-label, phase 2 trial. Lancet Oncol..

[35] Le Cesne A., Marec-Berard P., Blay J.Y., Gaspar N., Bertucci F., Penel N., Bompas E., Cousin S., Toulmonde M., Bessede A. (2019). Programmed cell death 1 (PD-1) targeting in patients with advanced osteo-sarcomas: Results from the PEMBROSARC study. Eur. J. Cancer..

[36] Recommendations for Treatment with Immune Checkpoint Inhibitors in Sarcoma. http://www.ssg-org.net/treatment-protocols-and-recommendations/ongoing.

